# Fabrication and Properties of Hydrogel Dressings Based on Genipin Crosslinked Chondroitin Sulfate and Chitosan

**DOI:** 10.3390/polym16202876

**Published:** 2024-10-11

**Authors:** Ling Wang, Xiaoyue Ding, Xiaorui He, Ning Tian, Peng Ding, Wei Guo, Oseweuba Valentine Okoro, Yanfang Sun, Guohua Jiang, Zhenzhong Liu, Armin Shavandi, Lei Nie

**Affiliations:** 1 College of Life Sciences, Xinyang Normal University, Xinyang 464000, China; wangling@xynu.edu.cn (L.W.); dingxiaoyue123456@126.com (X.D.); hexiaorui2021@163.com (X.H.); ningtian5112003@163.com (N.T.); dingzhiyu120@163.com (P.D.); weiguo@xynu.edu.cn (W.G.); 2 3BIO-BioMatter, École Polytechnique de Bruxelles, Université libre de Bruxelles (ULB), Avenue F.D. Roosevelt, 50-CP 165/61, 1050 Brussels, Belgium; oseweubaokoro@gmail.com (O.V.O.); amin.shavandi@ulb.be (A.S.); 3 College of Life Sciences and Medicine, Zhejiang Sci-Tech University, Hangzhou 310018, China; katherineyfs@zstu.edu.cn; 4 School of Materials Science and Engineering, Zhejiang Sci-Tech University, Hangzhou 310018, China; ghjiang_cn@zstu.edu.cn; 5 International Scientific and Technological Cooperation Base of Intelligent Biomaterials and Functional Fibers of Zhejiang Province, Zhejiang Sci-Tech University, Hangzhou 310018, China; 6 Taizhou Key Laboratory of Medical Devices and Advanced Materials, Taizhou Institute of Zhejiang University, Taizhou 318000, China

**Keywords:** genipin, chitosan, chondroitin sulfate, interpenetrated network, hydrogel, wound dressings

## Abstract

Multifunctional hydrogel dressings remain highly sought after for the promotion of skin wound regeneration. In the present study, multifunctional CHS-DA/HACC (CH) hydrogels with an interpenetrated network were constructed using hydroxypropyl trimethyl ammonium chloride modified chitosan (HACC) and dopamine-modified chondroitin sulfate (CHS-DA), using genipin as crosslinker. The synthesis of HACC and CHS-DA was effectively confirmed using Fourier transform infrared (FT-IR) analysis and ^1^H nuclear magnetic resonance (^1^H NMR) spectroscopy. The prepared CH hydrogels exhibited a network of interconnected pores within the microstructure. Furthermore, rheological testing demonstrated that CH hydrogels exhibited strong mechanical properties, stability, and injectability. Further characterization investigations showed that the CH hydrogels showed favorable self-healing and self-adhesion properties. It was also shown that increasing HACC concentration ratio was positively correlated with the antibacterial activity of CH hydrogels, as evidenced by their resistance to *Escherichia coli* and *Staphylococcus aureus*. Additionally, Cell Counting Kit-8 (CCK-8) tests, fluorescent images, and a cell scratch assay demonstrated that CH hydrogels had good biocompatibility and cell migration ability. The multifunctional interpenetrated network hydrogels were shown to have good antibacterial properties, antioxidant properties, stable storage modulus and loss modulus, injectable properties, self-healing properties, and biocompatibility, highlighting their potential as wound dressings in wound healing applications.

## 1. Introduction

Wound healing is a multifaceted physiological process that facilitates repairing and regenerating damaged skin tissue. Wound dressing constitutes the key medical treatment employed to accelerate wound healing by limiting wound injury and bacterial invasion [1,2]. Traditional wound dressings, which are usually based on foam, membranes, and gauze or gauze sheets, do not provide optimal conditions for healing since they may cause secondary damage during their removal [3]. There is, therefore, a need to develop novel wound dressings, with hydrogels identified as capable of functioning as a biomaterial platform to satisfy critical wound dressing requirements such as biocompatibility and ease of installation and removal for painless dressing changes. Crucially, the structure of hydrogels supports the inherent capacity to absorb wound exudates while simultaneously maintaining a moist environment [4,5]. These hydrogels can also be infused with anti-inflammatory or bioactive substances that are gradually released into the wound, thereby accelerating the healing process.

Chondroitin sulfate (CHS) has been identified as a significant potential anti-inflammatory agent [6]. CHS possesses a variety of important biological activities, including anti-inflammatory effects, absorption of nutrients and water, regulation of joint metabolism, and the promotion of chondrocyte proliferation [6]. Due to its attractive characteristics, CHS has become a promising biomedical material [7,8]. However, CHS has some limitations, such as poor adhesion and difficulty forming solid structures, thus highlighting the need to integrate it with other materials for practical applications. For instance, the adhesive properties of CHS can be improved by modification using dopamine [9]. This is because dopamine contains L-dopa and lysine residues, which are prone to oxidative auto-polymerization under alkaline conditions, forming a tight polydopamine coating on any surface for enhanced adhesion properties [8]. Furthermore, the antibacterial activity of dressing plays an essential role in promoting wound healing. Chitosan, another natural polysaccharide polymer consisting of N-acetylglucosamine and D-glucosamine monomer, has good antibacterial, hemostatic, and biocompatibility properties. However, the poor water solubility of chitosan makes it difficult to be applied in the field of biomedicine. Chitosan modified with quaternary ammonium salt has better solubility and antibacterial properties and can be used as a good source of wound dressing [10,11]. Chemical crosslinking enhances hydrogel mechanical properties. Genipin, superior to traditional agents like glutaraldehyde, offers excellent biocompatibility and can crosslink with various biomedical materials, including proteins and chitosan. This suitability extends to artificial bone, wound dressings, and other applications [12]. Moreover, genipin improves mechanical properties and stability without altering the material’s microstructure and biological characteristics [12].

**Scheme 1:**
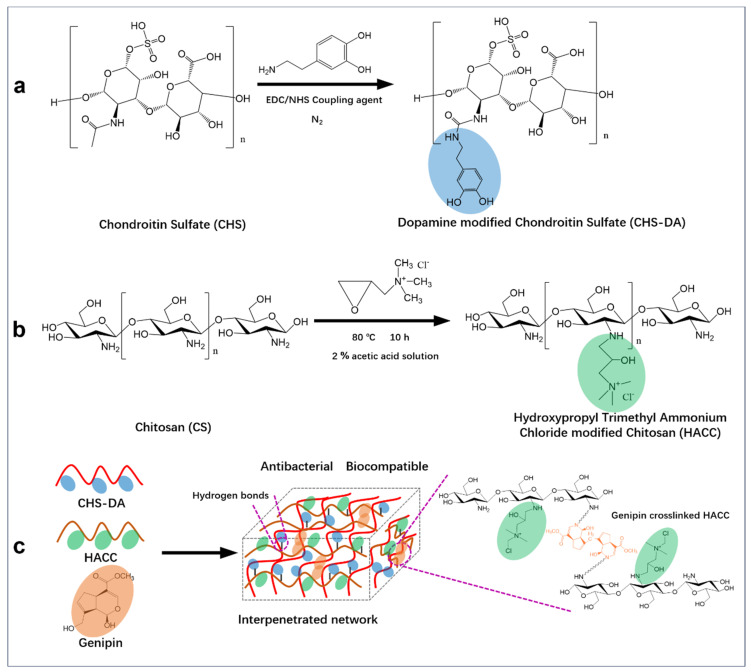
The schematic representation of CH hydrogel preparation: (**a**) Synthesis of dopamine-modified chondroitin sulfate (CHS-DA), (**b**) Production of hydroxypropyl trimethyl ammonium chloride-modified chitosan (HACC), and (**c**) Fabrication of CH hydrogel with an interpenetrated network structure via genipin crosslinking polymers of HACC and CHS-DA.

Considering this context, this present study prepared a novel hydrogel with an interpenetrated network based on genipin crosslinked chitosan and chondroitin sulfatefor potential application in wound dressings. As shown in Scheme 1, the dopamine-modified chondroitin sulfate was crosslinked with quaternary aminated chitosan using a green method, with genipin as a crosslinking agent. By a chemical modification of crosslinking agents, the antigenicity of hydrogel dressing to the wound is reduced, and their ability to resist mechanical change and degradation of the recipient tissue is simultaneously improved. In addition, based on the dopamine modification on CHS, quaternary aminated chitosan crosslinked using genipin, the multi-functional hydrogel dressings were fabricated.

## 2. Materials and Methods

### 2.1. Materials

Chitosan and N-hydroxy succinimide (NHS) were sourced from Macklin Biochemical Co, Ltd. (Shanghai, China) Chondroitin sulfate (CHS), ethyl alcohol, morpholine ethane sulfonic acid (MES), and N-(3-Dimethylaminopropyl)-N’-ethylcarbodiimide hydrochloride (EDC) were obtained from Ron Reagents Co, Ltd. (Shanghai, China) 2,3-cyclochloropropul trimethyl ammonium chloride (GTMAC) was purchased from Bide Pharmatech Co., Ltd. (Shanghai, China) Dopamine was sourced from Aladdin Biochemical Technology Co., Ltd. (Shanghai, China) Genipin was acquired from Energy Chemical Co., Ltd. (Shanghai, China) Acetic acid was procured from Sinopharm Chemical Reagent Co., Ltd. (Shanghai, China) Ultrapure water was obtained using a Milli-Q50 SP water system (Millipore Corporation, Billerica, MA, USA). All chemicals were used as received without any further purification.

### 2.2. Preparation of Dopamine-Modified Chondroitin Sulfate (CHS-DA)

Dopamine-modified chondroitin sulfate (CHS-DA) was prepared using a method described in the literature [13]. The morpholine ethane sulfonic acid (MES) buffer was initially prepared by introducing 1.95 g MES in 100 mL Millipore water, with the solution’s pH adjusted using ammonium hydroxide solution to 5.0. After that, 400 mg chondroitin sulfate was introduced in 80 mL of the buffer of MES, with the solution magnetically stirred while maintaining a nitrogen (N_2_) atmosphere. The solution was stirred for 30 min to obtain solution A. Next, 437 mg EDC and 495 mg NHS were added to 2 mL of the MES buffer. The mixture was then introduced into solution A. The mixed solution was stirred for 30 min, and then a 2 mL of MES buffer 432.4 mg DA, wholly dissolved, was added, and the mixed solution was magnetically stirred for 12 h at room temperature. Subsequently, dialysis was carried out for 5 days in Millipore water while using a dialysis membrane with a molecular weight cutoff of 3500 Da, with the water being replaced 3 times daily. After dialysis, the product was placed in a −20 °C freezer for 1 day and freeze-dried to obtain dried CHS-DA. The sample was kept in a refrigerator at 4 °C until needed.

### 2.3. Preparation of Hydroxypropyl Trimethyl Ammonium Chloride Modified Chitosan (HACC)

Based on our previous works, the hydroxypropyl trimethyl ammonium chloride modified chitosan (HACC) was synthesized [14,15]. In summary, 8.0 g of chitosan and 7.2 g of 2,3-epoxypropyl trimethyl ammonium chloride were mixed in 300 mL Millipore water in a flask. The reaction was under N_2_ protection in the three-necked flask: one neck for N_2_ input, another neck for N_2_ output, and the middle neck for reflux. The solution was refluxed for 36 h at 80 °C. The homogeneous solution was dialyzed in Millipore water at room temperature for 5 days, with water changes occurring at least three times daily to remove impurities. The dialyzed solution was subsequently freeze-dried to yield a solid product (HACC).

### 2.4. Fabrication of Interpenetrated Network CHS-DA/HACC (CH) Hydrogels

The CH hydrogel with an interpenetrated network was fabricated by mixing CHS-DA and HACC solutions using genipin as a crosslinker [16]. CHS-DA was dissolved in Millipore water to prepare 2 *w*/*v*% and 4 *w*/*v*% solutions. The HACC was introduced into the 2 *v*/*v*% acetic acid to prepare 3 *w*/*v*% and 6 *w*/*v*% solutions. At the same time, 0.0018 g genipin was dissolved in 0.1 mL ethyl alcohol. After that, CHS-DA solution, HACC solution, and genipin solution were mixed, and the CH hydrogel could be obtained within 3 h, resulting in a sticky, dark hydrogel. Based on the weight ratios of CHS-DA/HACC at 1, 2/3, and 3/4, CH1, CH2, and CH3 hydrogels were prepared accordingly (Table 1).

### 2.5. H Nuclear Magnetic Resonance (^1^H NMR) Test

The prepared polymers CHS-DA and HACC were tested using a ^1^H nuclear magnetic resonance spectrometer (^1^H NMR, 600 MHz, JEOL Ltd., ECZ600R/S3, Musashi Murayama, Japan) to establish their successful synthesis. The sample was dissolved in D_2_O and then transferred to a standard NMR tube, and the liquid level in the tube was 4 cm. Following that, the ^1^H NMR spectrum of CHS-DA and the ^1^H NMR spectrum of HACC were recorded.

### 2.6. Fourier Transform–Infrared Spectrometer (FT-IR) Test

To further evaluate the functional groups grafted to the synthesized polymers CHS-DA and HACC, a Fourier transform–infrared spectrometer (FT-IR, Bruker, VERTEX 70, Bremen, Germany) was employed. The dried potassium bromide (KBr) was mixed with the tested sample, and the thin round disc was obtained by pressing. The FT-IR spectra were then recorded over the wavelength range of 500–4000 cm^−1^ with a resolution of 1 cm^−1^, and each spectrum was taken with 64 scans.

### 2.7. Scanning Electron Microscopy (SEM) Test

The CH hydrogels were examined using cold field emission scanning electron microscopy (SEM, Hitachi S-4800, Chiyoda-ku, Tokyo, Japan). To undertake this assessment, the lyophilized hydrogels were initially immersed in liquid nitrogen and then fractured. Before SEM observation, a platinum layer was sputter-coated on the surface of the samples for 40 s. Five SEM images were generated for each sample, and the pore size distribution was subsequently determined using ImageJ software (1.54g).

### 2.8. Mechanical Stress–Strain Test of CH Hydrogels

The CH hydrogels were tested for their mechanical properties using a universal testing apparatus (CMT-4103, Zhuhai Junjie Technology Co., Ltd., Zhuhai, China) equipped with a 25 mm diameter cylindrical probe and a 1 kN load cell [14]. The hydrogel samples were molded into cylindrical shapes with a diameter of 10 mm and a height of 10 mm. Stress–strain data were collected during compression testing performed at a speed of 0.007 mm/s.

### 2.9. Rheological Evaluation

A TA rheometer (DHR-2, TA Instruments Co., Ltd., New Castle, DE, USA) was employed to evaluate the CH hydrogel’s rheological properties at room temperature. All tested samples were prepared in a circular spline of a 25 mm diameter, and glycerin was used to seal the edge of the fixture for the prohibition of water evaporation during the test. First, the storage modulus (G′) and loss modulus (G″) of CH hydrogels were recorded by varying the strain (0.1–600%) while keeping the frequency at 1 Hz to determine the linear viscoelastic region of the hydrogels. After the linear viscoelastic zone was confirmed, G′ and G″ were recorded in the range of 0.1–15 rad/s at room temperature. A time test was conducted from 0 to 300 s with a frequency of 1 Hz and a constant strain of 1%.

### 2.10. The Adhesive and Self-Healing Properties Analysis

Hydrogels’ adhesive properties are crucial for their wound dressing applications, with the present study evaluating the adhesive properties of CH hydrogels. The CH hydrogel samples were applied to the surface of various matrices, including human skin, rubber, plastic, wood, metal, and glass. The adhesion results were recorded through photographs, showcasing the hydrogels’ effectiveness in adhering to these different materials. Next, the self-healing capabilities of the hydrogels were assessed. To this end, the macroscopic method was employed. Briefly, each hydrogel sample was sliced into two sections, which were then placed together in a petri dish and left in contact at room temperature for 1 min. Photographs were taken to evaluate the self-healing potential of the hydrogels [17]. Additionally, the self-healing capacity was quantitatively assessed using a modular compact rheometer. A strain amplitude scan test (γ = 0.1–600%) was conducted at 25 °C with a constant angular frequency of 10 rad/s, and the critical strain region was recorded [18,19].

### 2.11. Injectability Analysis

The injectable ability of CH hydrogels was evaluated using direct extrusion by a syringe, and viscosities over shear rate were measured using a rheometer [20]. The CH hydrogel was loaded into a 2.5 mL syringe and then extruded. The alphabets were written using the syringe to display the injectability of CH hydrogels, and corresponding photos were recorded. Additionally, the hydrogels’ injectibility was assessed by examining how viscosity varies with shear rate using a rheometer. Oscillation–frequency tests were performed at a constant strain of 1%, with the shear rate ranging from 0.1 rad/s to 100 rad/s at 25 °C [14].

### 2.12. Equilibrium Swelling Analysis

To determine the equilibrium swelling ratios of the CH hydrogels, the mass of the CH hydrogel sample was first measured and recorded (*W_A_*). The sample was introduced to the PBS solution (pH 7.4) at 37 °C, and the mass of the hydrogel was measured at 0.5 h intervals. Before weighing, the water on the surface of the hydrogel was removed using a filter paper. While the weight of the water-absorbed hydrogel was kept in an equilibrium state, the weight was recorded as *W_B_*. The equilibrium swelling ratio (%) of the samples was subsequently calculated as follows [21]:

$$SR\;\left(\%\right)=\frac{W_{B}-W_{A}}{W_{A}}\times 100\;\%$$

### 2.13. Antioxidant Test

The antioxidant activity of CH hydrogels was assessed using the 1,1-diphenyl-2-picrylhydrazyl (DPPH) free-radical scavenging assay [22]. Briefly, a 15 mg hydrogel sample was introduced in a mixed DPPH/ethanol solution (1 mL ethanol with 0.1 mM DPPH solution), and then the mixture was incubated in a dark tube for 30 min. After that, the absorption of the mixture (supernatant) at 517 nm was detected using an enzyme marker (Synergy H1, Bio Tek, Shanghai, China). The control group was set up without adding any hydrogel sample. The DPPH radical scavenging rate was then calculated using the following equation:

$$DPPH\;Scavenging\;rate\;\left(\%\right)=\frac{A_{0}-A_{1}}{A_{0}}\times 100\;\%$$

where *A*_0_ denotes the absorbance of the control group, and *A*_1_ represents the sample group.

### 2.14. Antibacterial Analysis

The in vitro antibacterial activity of the CH hydrogels was evaluated using a standard antibacterial test method outlined in the literature, with *S. aureus* and *E. coli* serving as the test bacterial strains [23,24]. *S. aureus* (ATCC 6538) and *E. coli* (ATCC 25922) were obtained from ATCC company. Briefly, the hydrogel samples with a uniform thickness of 4 mm were prepared and irradiated under UV light for 30 min. A 100 μL suspension of *E. coli* (5 × 10^7^ CFU mL^−1^) and *S. aureus* (6 × 10^7^ CFU mL^−1^) were evenly spread on Luria-Bertani (LB) solid agar plates. The hydrogel samples were added on solid plates that had been coated with a bacterial suspension. Incubation of the plates was then undertaken at 37 °C for 8 h. Following incubation, the size of the inhibition zones around each sample was measured with a vernier caliper, and the diameter was determined by conducting each test in triplicate.

### 2.15. Biocompatibility Evaluation

The mouse embryonic fibroblast cells (NIH-3T3 cells, CRL-1658^TM^, obtained from ATCC company, Manassas, WV, USA) were used to test the biocompatibility of CH hydrogels [25]. To this end, the cells (1 × 10^5^ per well, 100 μL) were inserted in 96-well plates with Dulbecco’s Modified Eagle Medium (DMEM) supplemented with 10% fetal bovine serum (FBS) and 1% penicillin-streptomycin solution. The plates were then incubated in a humidified environment with 5% CO_2_ at 37 °C. Cells cultured in the absence of hydrogels were used as the control group. Hydrogel extracts were utilized and prepared for the cell culture according to the procedure described below [26]. The freeze-dried hydrogels were put into centrifuge tubes, followed by the addition of 75 *v/v*% alcohol for soaking and sterilization for 12 h, after which PBS solution (pH = 7.4) was used in washing the hydrogels five times. Next, the sample was immersed in DMEM medium (without FBS) and incubated at 37 °C for 24 h to prepare the hydrogel extract, and the sample/medium ratio was around 0.1 g/mL. After that, NIH-3T3 cells were inoculated into a 96-well plate containing the hydrogel extracts. After culturing in a humid incubator containing 5% CO_2_ at 37 °C for 1, 3, and 5 days, 10 μL of CCK-8 reagent was added to each well and incubated for 2 h. Absorbance at 450 nm was measured using a microplate reader (SpectraMax 190, Molecular Devices, Silicon Valley, CA, USA). Furthermore, the cell morphologies of the co-cultured cells with hydrogel extracts were examined at 1, 3, and 5 days using fluorescent microscopy. Cells were stained using Calcein-AM/PI (live/dead) to evaluate their durability and morphology.

### 2.16. Cell Migration Assay

Cell migration was studied using NIH-3T3 cells that were cultured with hydrogel extracts. The NIH-3T3 cells were inserted in a 96-well plate and grown until a monolayer of cells covered the bottom of each well. A 10 uL pipette sharp tip was employed to scratch a straight line across the cell monolayer. After cleaning with PBS solution, the initial scratch photo was taken under an inverted microscope. Afterwards, the same amount of hydrogel extract was introduced to each well, after which culturing of the cells was undertaken for 8 h. The washing of the cells was then undertaken using PBS, and the scratch photos were taken; ImageJ software (1.54g version) was used in calculating the migration rate as follows:

$$Migration\;rate\;\left(\%\right)=\frac{A_{0}-A_{8}}{A_{0}}\times 100\;\%$$

where *A*_0_ indicates the initial blank area (i.e., at time = 0 h), and *A*_8_ represents the blank area at 8 h.

### 2.17. Statistical Analysis

The results are presented as means ± standard deviation (SD). Statistical analysis was performed using ANOVA and Tukey’s test on SPSS software (Version 26.0, SPSS Inc., Chicago, IL, USA), with significance levels set at *p*-values of <0.05, <0.01, and <0.001, corresponding to confidence levels of 95%, 99%, and 99.9%, respectively. Data plotting was performed using Origin software (Origin 2021 version), and ANOVA was used for data analysis.

## 3. Results and Discussion

### 3.1. Synthesis of CHS-DA and HACC

**Figure 1:**
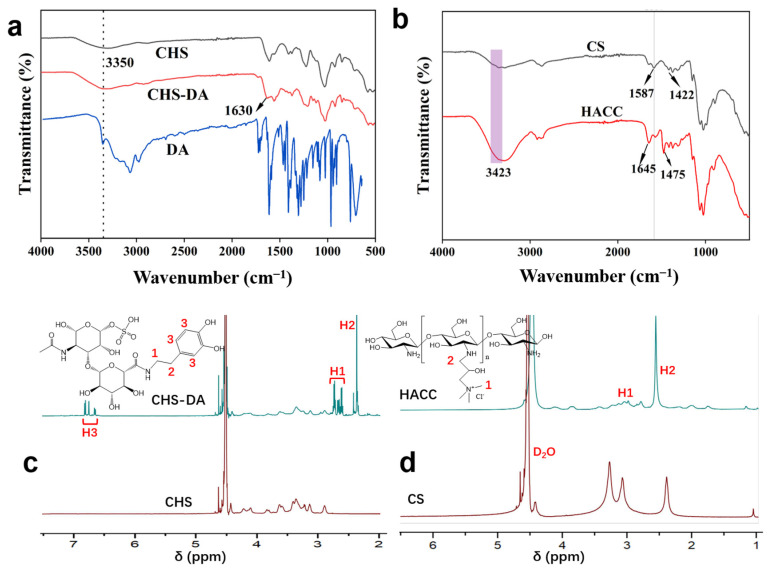
(**a**) Chondroitin sulfate (CHS), DA, and CHS-DA FTIR spectra. (**b**) ^1^H NMR spectra comparing CHS with the synthesized CHS-DA. (**c**) Chitosan and HACC FT-IR spectra. (**d**) The spectra (^1^H NMR) of chitosan and the produced HACC.

Before fabricating the CH hydrogels, CHS-DA and HACC were prepared, and their successful synthesis was confirmed using ^1^H-NMR analysis and FT-IR tests. Figure 1a shows that the FT-IR spectra of CHS have a broad band at around 3436 cm^−1^, which is due to the overlap of -OH and N-H stretching, and the typical peaks at 1037 cm^−1^ and 925 cm^−1^ were observed, which are attributed to the stretching of C-O and α-(1→4) glucosidic bonding [27]. In addition, new peaks at the 3350 cm^−1^ and 1630 cm^−1^ peaks attributed to the stretching vibration of the hydroxy group from dopamine and imine bonds (-CO-NH-), respectively, were detected in the spectrum of CHS-DA, confirming the successful synthesis of CHS-DA. In the ^1^H NMR spectrum of CHS-DA (Figure 1b), the peaks at 3.3 ppm, 2.8 ppm, and 7.3 ppm are characteristic of alkyl protons (1) and (2) in the dopamine residues, and the aromatic matrix (3), respectively, further verifying the successful CHS-DA synthesis. At the same time, HACC was obtained using the typical chemical modification method according to our previous works [14,15,25]. In the spectrum of CS (Figure 1c), peaks were detected at 3423 cm^−1^ and 1572 cm^−1^ and corresponded to the vibration of the primary amine (-NH_2_) functional group. The peak observed at 1422 cm⁻^1^ was attributed to the stretching vibration of the C-N bond. In the FT-IR spectrum of HACC, the distinctive peak at 1475 cm⁻^1^ was associated with the C-H bond. In the trimethylammonium group of GMTAC and a dominant peak at 1645 cm^−1^ was observed, which is associated with the N-H bond bending, indicating the successful quaternary ammonium group graft to chitosan chain. In addition, ^1^H NMR was further used to analyze the synthesis of HACC and the quaternary ammonium group substitution degree. The ^1^H NMR spectra of HACC (Figure 1d) detected two peaks at 3.2 and 3.5 ppm, corresponding to the trimethylammonium and -NH-CH2- groups, respectively. Additionally, the degree of substitution of the quaternary ammonium group in HACC was determined to be 3.02 ± 0.21% based on the ∫(N^+^(CH_3_)_3_)/∫(H_1_) ratio obtained from the ^1^H NMR spectra of HACC.

### 3.2. CH Hydrogels Production

**Figure 2:**
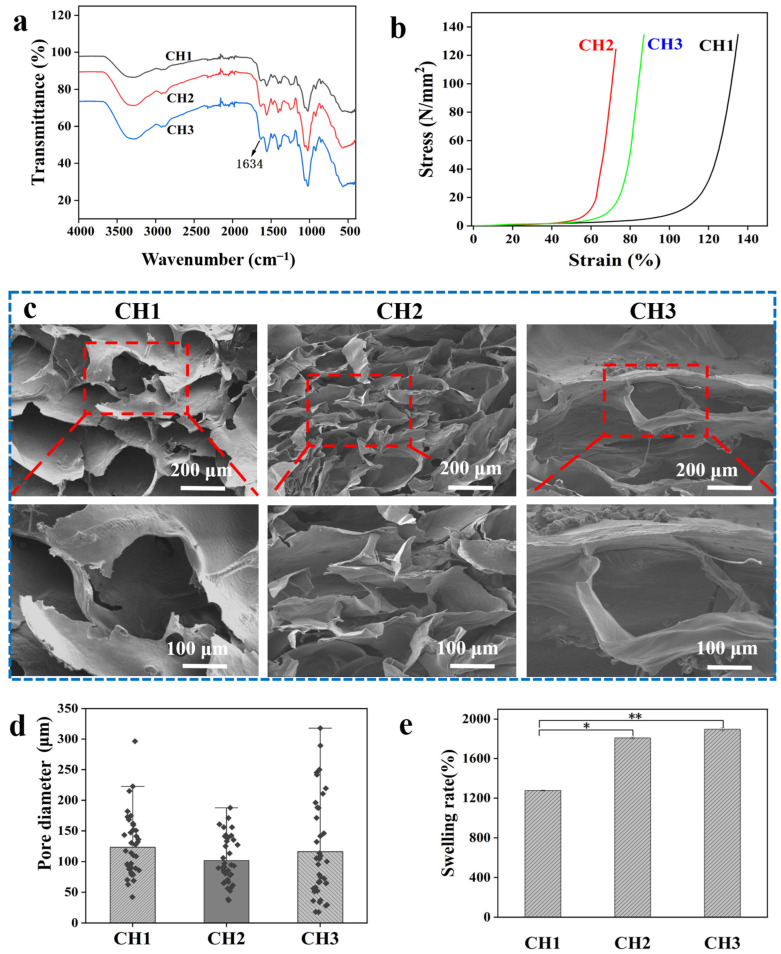
Characterization of the synthesized CH hydrogels: (**a**) FT-IR spectra of CH hydrogels. (**b**) Stress–strain profiles of CH hydrogels. (**c**) SEM images of CH hydrogels at various magnifications. (**d**) Pore size distribution of CH hydrogels was calculated from SEM images using ImageJ software (1.54g version). (**e**) Equilibrium swelling ratio of CH hydrogels in PBS. * *p* < 0.05 and ** *p* < 0.01.

Based on the synthesized CHS-DA and HACC, CH hydrogels with an interpenetrated network structure were fabricated, in which a CHS-DA polymer chain formed the first network, and genipin crosslinked HACC polymer chain formed the second network (Scheme 1c). The HACC polymer chain was crosslinked using genipin with the Dipole–Dipole force and Coulomb force [28]. The interactions of polymers present in CH hydrogels were investigated using FT-IR, and the FT-IR spectra of CH hydrogels are displayed in Figure 2a. Besides the observed characteristic peaks associated with CHS-DA and HACC, CH1, CH2, and CH3 hydrogels presented similar transmittance spectrum. Furthermore, the characteristic peak at 1634 cm^−1^ for the vibration of the amide band (-C=N-) was observed and confirmed that Schiff base bonds occurred in CH hydrogels. In our previous work, we confirmed that the dimer bridges could be formed during the genipin crosslinking HACC process, and the terminal aldehyde groups further resulted in the formation of Schiff base links [14,29]. The formed network of the dynamic Schiff base crosslinked HACC could further influence the hydrogel’s mechanical properties. The mechanical properties are important to the application scenarios of hydrogels. A universal mechanical testing machine was used to measure the stress of CH hydrogels, as shown in Figure 2b. For CH1, CH2, and CH3 hydrogels, the weight ratios of HACC/CHS-DA were 1, 3/2, and 4/3, respectively. With the increase in the weight ratio of HACC/CHS-DA, CH hydrogels exhibited increasing stress at the same strain point, indicating improved stress performance [30].

### 3.3. Microstructure and Swelling Properties

Scanning electron microscopy (SEM) was used to analyze the morphology and microstructure of the CH hydrogels, offering valuable insights into how their porous architecture influences water absorption capacity. In this regard, Figure 2c shows that all fabricated CH hydrogels exhibit interconnected pore structures that can support the absorption of excess wound exudate and promote oxygen transfer, thereby promoting wound healing [31]. Based on the obtained SEM images, pore size distribution was determined via ImageJ software (1.54g version) (Figure 2d). Compared to hydrogels CH3 and CH1, the CH2 hydrogel with the highest HACC ratio possessed a smaller pore size. In the mechanical test, the CH2 hydrogel displayed the highest stress performance. It was determined that the hydrogel’s mechanical stress was inversely related to the microstructure. In addition, the swelling ability of a hydrogel is greatly influenced by its microstructure [32]. When applied to a wound, a hydrogel’s swelling ability is crucial for absorbing exudates and retaining the water in the network, so that the wound environment remains moist and promotes the healing of the epithelial tissue [31]. This characteristic is a significant feature of a wound dressing based on a hydrogel. The equilibrium swelling ratio of CH hydrogels was analyzed; the results are shown in Figure 2e. Among the samples, CH3 exhibited the highest swelling rate, reaching 2004.5%, whereas CH1 showed the lowest swelling rate at 1244.6%. The rate of swelling of composite hydrogels increases as the content of HACC in the CH hydrogels increases. This contrast indicates that the interconnected network structure and microporous architecture are more conducive to water absorption.

### 3.4. Rheological and Injectability Characteristics

**Figure 3:**
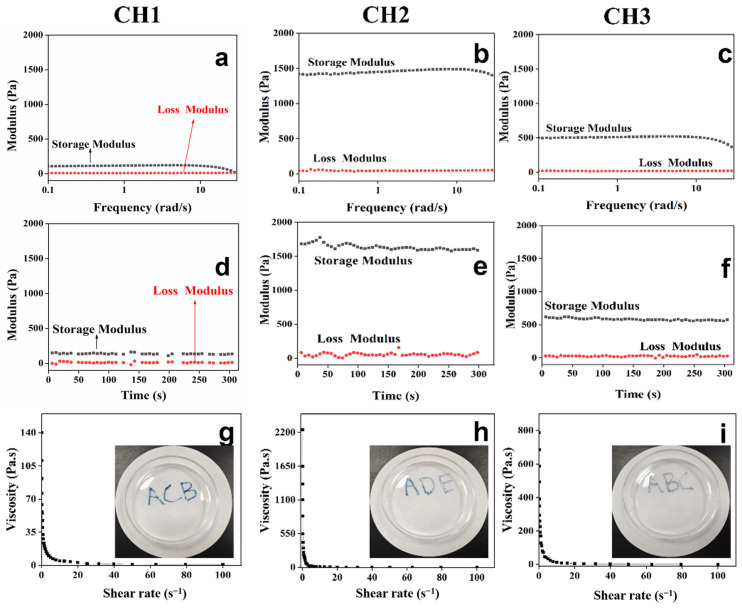
Rheological analysis of the CH hydrogels: the storage modulus (G′) and loss modulus (G″) were measured as functions of frequency (**a**–**c**) and time (**d**–**f**). In addition, the frequency was kept at 1 Hz during G′ and G″ testing over time. Panels (**g**–**i**) show the variations in viscosity of the CH hydrogels with shear rate, and viscosity could reflect a fluid’s resistance to a change in shape or movement. The inset images illustrate that the CH hydrogels can be injected using a syringe.

The rheological characteristics of the prepared CH hydrogels are shown in Figure 3. The viscoelasticity of each CH hydrogel was tested using a 1% constant-strain frequency scan. The results indicated that each hydrogel’s storage modulus (G′) exceeded the loss modulus (G″), demonstrating that all CH hydrogels exhibited strong viscoelastic properties. Rheological mechanical tests were also performed to evaluate the mechanical characteristics of the hydrogel for wound applications. Figure 3a–f show that the storage modulus (G′) of CH1, CH2, and CH3 hydrogels were 132 Pa, 1597 Pa, and 571 Pa, respectively, across both the time and frequency domains. Notably, the CH hydrogels showed relatively stable G′ and G″ values over frequency and time. The enhanced mechanical properties of CH hydrogels can be ascribed to the strengthened bonds between the -OH and -COOH functionalities of genipin and the amino functionalities of HACC, which lead to improved crosslinking [14]. This enhanced crosslinking leads to better mechanical strength and stability, making CH hydrogel a promising candidate for wound dressing applications. Analyses of the injectable characteristics of CH hydrogels, strain scan rheological tests and apparent tests were performed. As viscosity is defined as the resistance of a fluid to changes in the shape or movement of neighboring portions relative to one another, the injectability property of the hydrogel could be assessed based on the viscosity parameter of the rheometer (Figure 3g,h). Figure 3g,h show that the viscosities of CH hydrogels present an inverse relation with increasing shear rate, highlighting their shear-thinning behavior. Thus, CH hydrogels may be readily injected from a syringe. Different letters such as “ACB”, “ADE”, and “ABC” were written using CH1, CH2, and CH3 hydrogels, respectively. The letters in light blue kept the shape fidelity, confirming the excellent injectability of CH hydrogels. Furthermore, good injectability, which allows for the hydrogel to be filled into the irregular wound, was assessed [33] with no blockage observed when the extrusion operation was performed using a syringe with a 2.5 mL diameter.

### 3.5. Self-Healing and Adhesive Properties

**Figure 4:**
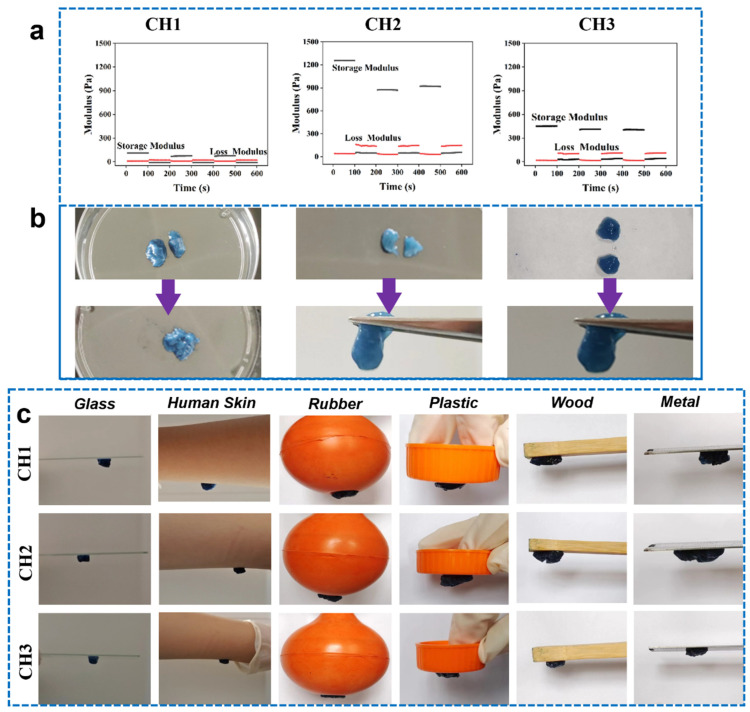
(**a**) A three-step cyclic strain test was conducted using a rheometer to assess the self-healing capabilities of the CH hydrogels. (**b**) The self-healing properties of the CH hydrogels were further examined through macroscopic observation, where the hydrogels were cut into two pieces and rejoined, and their contact morphology was documented. (**c**) Images showing the adhesion of CH hydrogels to various substrates, including glass, human skin, rubber, plastic, wood, and metal.

The self-healing characteristic of hydrogel dressings could facilitate their wound healing application in the clinic [34] and can be enhanced by the presence of hydrogen bonds in the hydrogel and dynamic crosslinking density. The self-healing characteristics of CH hydrogels were achieved quantitatively by applying alternating stress from 1% to 100% on the rheometer [18]. At 1% strain, the G′ values of the CH hydrogels were notably higher than the G″ values. However, when subjected to a high strain of 100%, the relationship was reversed (G″ > G′), indicating that the network structure of the CH hydrogels was compromised, preventing the formation of a hydrogel-like structure at that point. The cycling test results highlighted the CH hydrogels’ good self-healing ability. In addition, the self-healing ability was further confirmed using a macroscopic approach [35]. A hydrogel sample was cut into two pieces, which were then brought into contact for 1 min. The contact morphology of the hydrogel was subsequently assessed. Figure 4b shows that all the CH hydrogels had good self-healing ability; the CH2 and CH3 hydrogels displayed a better self-healing behavior than the CH1 hydrogel, which is mainly due to the higher dynamic crosslinking density in the CH2 and CH3 hydrogels than the CH1 hydrogel. In addition, the CH hydrogels were also observed to show their adhesion properties on various surfaces, including glass, skin, rubber, plastic, wood, and metal (Figure 4c). The adhesive properties of fabricated CH hydrogels were mainly due to the positively charged amino group in HACC, which engages in electrostatic interaction with the skin surface as well as hydrophobic interaction [12].

### 3.6. Antibacterial and Antioxidant Properties

**Figure 5:**
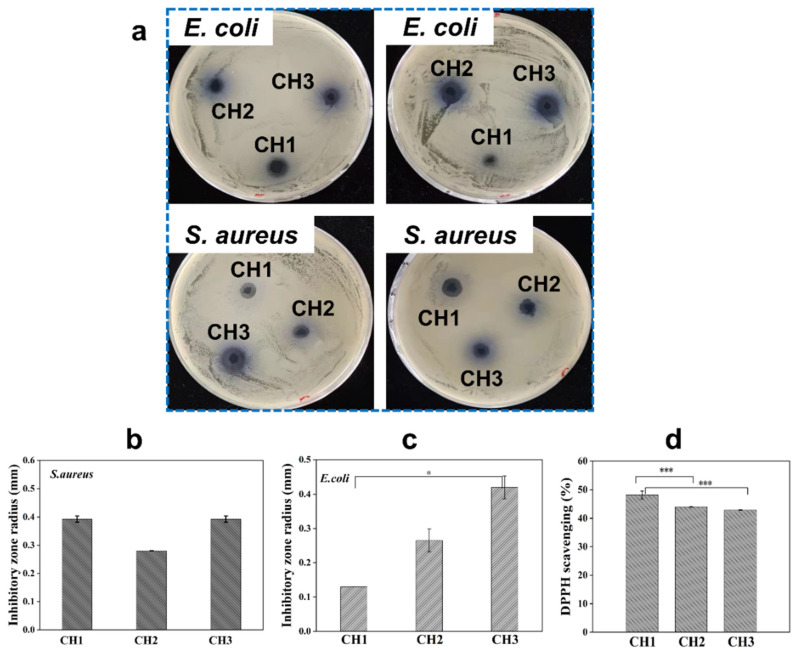
(**a**) Images showing the inhibition zones created by CH hydrogels against *E. coli* and *S. aureus* after 8 h, demonstrating their antibacterial properties. Note that the left and right images represent repeated experiments. (**b**,**c**) The radius of the inhibition zones for CH hydrogels against (**b**) *S. aureus* and (**c**) *E. coli*, calculated based on the observed zones. (**d**) DPPH radical scavenging rate of CH hydrogels within 30 min, highlighting their antioxidant activity. * *p* < 0.05 and *** *p* < 0.001.

Inflammation caused by bacterial infection can delay wound healing. Hydrogels with antibacterial properties reduce the probability of bacterial infection, thus minimizing the need for antibiotics [36]. Solid antibacterial experiments were undertaken using typical bacteria, *E. coli* and *S. aureus*, to assess the antibacterial activities of the hydrogel*s*. Figure 5a–c show that the inhibitory zones were clearly observed for all CH hydrogels after culturing with *S. aureus* and *E. coli* for 8 h, indicating that the CH hydrogels possess antibacterial properties. This may be attributed to the electrostatic attraction between the positively charged amino groups in HACC and the negatively charged bacteria surfaces, with the interaction causing the rupture of the bacterial surface and leading to bacterial death [11,37]. The antibacterial effects of HACC against both *E. coli* and *S. aureus* were demonstrated, highlighting its prospect as an effective material for wound dressings. Such antimicrobial dressings can greatly minimize the risk of wound infections, promoting more efficient and rapid healing [38]. Indeed, antioxidant activity is essential in the wound healing process, as it helps manage the excessive production of reactive oxygen species (ROS) [39]. These ROS are highly active molecules crucial to wound healing signaling pathways, with excessive ROS recognized as detrimental since they are capable of causing cellular and tissue damage, accelerating ageing, and contributing to various degenerative diseases. Antioxidants are able to mitigate against the excessive ROS-induced damage by this counteracting effect by restoring enzyme function and enhancing metabolism, which supports cellular health and maintains redox balance within cells. The antioxidant properties of CH hydrogels were investigated using DPPH scavenging rate (Figure 5d). All CH hydrogels showed favorable DPPH scavenging rate (over 40%) in 30 min, and the sample CH1 hydrogel with the highest CHS-DA content displayed the higher scavenging rate compared to the CH2 and CH2 hydrogels. The CH hydrogels effectively reduced the peak intensity of DPPH by scavenging DPPH radicals, showcasing their strong antioxidant properties. This effect was probably a result of electron transfer from nitrogen ion groups or hydrogen atoms to the DPPH radicals [40].

### 3.7. Cytocompatibility Evaluation

**Figure 6:**
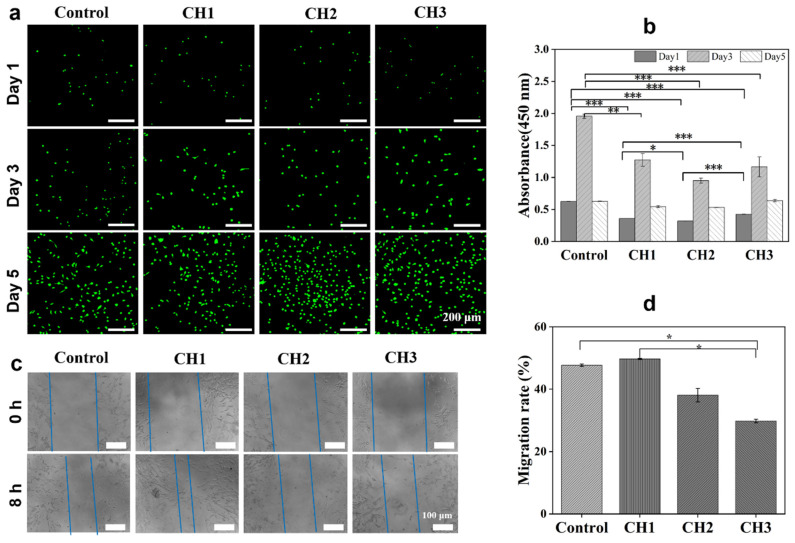
Biocompatibility and cell migration of CH hydrogels. (**a**) Fluorescent microscopy images of NIH-3T3 cells cultured with CH hydrogel extracts for 1, 3, and 5 days, respectively. Cells were stained with Calcein-AM/PI; scale bars: 200 μm. (**b**) Hydrogel cytocompatibilities were assessed with NIH-3T3 cells at various time points using the CCK-8 assay. (**c**) Scratch assay results for NIH-3T3 cells after 8 h of incubation, with blue dotted lines representing the width of the scratch gap. Scale bar: 100 μm. (**d**) The migration rate of NIH-3T3 cells based on scratch assay results. * *p* < 0.05, ** *p* < 0.01, and *** *p* < 0.001.

Excellent cytocompatibility is a prerequisite for designing hydrogel dressing in wound healing applications. In the present study, NIH-3T3 cells were used for culture with CH hydrogel extracts, and fluorescent microscopy images, CCK-8 analysis, and cell scratch were employed to investigate the cytocompatibility of CH hydrogels. After NIH-3T3 cells and the CH hydrogel extracts were co-cultured for 1 day, the cells showed a normal spindle shape, and it was clearly observed that the cell number in all groups increased over days, indicating that NIH-3T3 cells could grow and proliferate with a CH hydrogel extract (Figure 6a). The NIH-3T3 cell proliferation was quantitively evaluated using CCK-8, as shown in Figure 6b. It shows that the absorbance of each group was enhanced from day 1 to day 3, showing that the CH hydrogel extracts facilitated the proliferation of cells. However, the absorbance for all hydrogel groups and the control group decreased from day 3 to day 5. This phenomenon might be due to the over-growth of cells at day 3, which then results in decreased growth in the following days. The above results highlight the propagation of NIH-3T3 cells, highlighting the favorable biocompatibility of the CH hydrogels. Furthermore, the sample CH1 hydrogel exhibited a better cell growth rate than the CH2 and CH3 hydrogels, presenting better cytocompatibility due to the higher CHS-DA content in the CH1 hydrogel. With dopamine modification on CHS, the cell adhesion ability could be improved; thus, a higher CHS-DA content in hydrogels may promote cell growth and proliferation [41]. Next, the cell pro-migration ability of CH hydrogels was also investigated through the NIH-3T3 cell scratch test, and the results are shown in Figure 6c,d. Figure 6c shows that all CH hydrogels possessed cell migration ability after 8 h of co-culturing cells with hydrogel extracts, and the CH1 hydrogel exhibited a higher cell migration rate than the CH2 and CH3 hydrogels, which is in agreement with the CCK-8 results (Figure 6d). The results indicate that CH hydrogels could improve cell migration and potentially speed up the closure of cell scratches.

## 4. Conclusions

In this study, we fabricated a novel interpenetrated-network CH hydrogel based on dopamine-modified chondroitin sulfate and hydroxypropyl trimethyl ammonium chloride modified chitosan, using genipin as a crosslinker. The fabricated CH hydrogels with an interconnected microstructure exhibited favorable swelling and mechanical properties, injectability, self-healing ability, and adhesion properties. Furthermore, these multi-functional CH hydrogels displayed favorable antimicrobial ability. Based on the CCK-8 assay, fluorescent imaging, and cell scratch tests, the CH hydrogels demonstrated strong cytocompatibility and enhanced cell migration capabilities. The fabricated CH hydrogel dressings, incorporating chondroitin sulfate and chitosan crosslinked with genipin, demonstrated significant potential for wound care applications.

## Figures and Tables

**Table 1:** The composition of CHS-DA, HACC, and genipin for the fabrication of CH hydrogels.

Samples	^a^ CHS-DA Solution	^b^ HACC Solution	^c^ Genipin Solution	CHS-DA/HACC Weight Ratio
CH1	1.5 mL (4 *w*/*v*%, 0.06 g CHS-DA)	1 mL (6 *w/v*%, 0.06 g HACC)	0.1	1:1
CH2	1 mL (2 *w/v*%, 0.02 g CHS-DA)	1 mL (3 *w/v*%, 0.03 g HACC)	0.1	2:3
CH3	1.5 mL (2 *w/v*%, 0.03 g CHS-DA)	1.36 mL (3 *w/v*%, 0.04 g HACC)	0.1	3:4

^a^ CHS-DA was dissolved in Millipore water. ^b^ HACC was dissolved in a solution of acetic acid (2 *v*/*v*%). ^c^ 0.00018 g Genipin was dissolved in 0.1 mL ethyl alcohol.

## Data Availability

Data are contained within the article.
